# Analysis of the genetic diversity and mRNA expression level in porcine reproductive and respiratory syndrome virus vaccinated pigs that developed short or long viremias after challenge

**DOI:** 10.1186/s13567-018-0514-1

**Published:** 2018-02-15

**Authors:** Martí Cortey, Gaston Arocena, Tahar Ait-Ali, Anna Vidal, Yanli Li, Gerard Martín-Valls, Alison D. Wilson, Allan L. Archibald, Enric Mateu, Laila Darwich

**Affiliations:** 1 Departament de Sanitat i d’Anatomia Animals, Universitat Autònoma de Barcelona, 08193 Cerdanyola Del Vallès, Spain; 2 The Roslin Institute and Royal (Dick) School of Veterinary Studies, University of Edinburgh, Midlothian, EH25 9RG UK; 3 IRTA, Centre de Recerca en Sanitat Animal (CReSA, IRTA-UAB), Campus de la Universitat Autònoma de Barcelona, 08193 Cerdanyola Del Vallès, Spain

## Abstract

Porcine reproductive and respiratory syndrome virus (PRRSv) infection alters the host’s cellular and humoral immune response. Immunity against PRRSv is multigenic and vary between individuals. The aim of the present study was to compare several genes that encode for molecules involved in the immune response between two groups of vaccinated pigs that experienced short or long viremic periods after PRRSv challenge. These analyses include the sequencing of four SLA Class I, two Class II allele groups, and CD163, plus the analysis by quantitative realtime qRT-PCR of the constitutive expression of TLR2, TLR3, TLR4, TLR7, TLR8 and TLR9 mRNA and other molecules in peripheral blood mononuclear cells.

## Introduction, methods and results

### Introduction

Porcine reproductive and respiratory syndrome (PRRS) is one of the most economically important swine diseases worldwide [1]. Currently, two species of PRRS virus are recognized: PRRSv-1 and PRRSv-2 both within the genus *Porartevirus*, Family *Arteriviridae*. The genome size of PRRSv is about 15 Kb with at least 10 open reading frames (ORFs). ORF1a and 1b encode for two polyproteins that after enzymatic cleavage will result in at least 14 non-structural proteins (Nsps) involved in viral replication, plus at least two other proteins encoded after a ribosomal frameshift [2]. The 3′ end of the viral genome (ORFs 2a to 7) encodes five minor (GP2a, GP3, GP4, ORF5a protein and E) and three major (GP5, M and N) structural proteins [3].

The primary target of PRRSv are CD163^+^ macrophages that are common in lungs and lymphoid organs, particularly tonsil [4]. The entry of the virus into target cells involves: (i) an initial attachment to the cell surface, probably mediated by heparan sulfate (HS) [5] and porcine sialoadhesin-1 [6]—also known as CD169—or other sialoadhesins [7], probably by interacting with the GP5-M heterodimer, (ii) the internalization of the virus by endocytosis, (iii) the interaction with CD163, driven by the viral GP2-GP3-GP4-E complex, and (iv) the subsequent release of the viral genome into the cytoplasm [4]. Of the abovementioned receptors CD163 is considered to be the essential one. Actually, gene-edited pigs lacking the exon 7 of CD163 resulted in the generation of porcine alveolar macrophages (PAMs), and macrophages derived from peripheral blood completely resistant to both PRRSv-1 and 2 [8].

A key feature of PRRSv infection is the alteration of the host’s cellular and humoral immune response (reviewed by [9]). Typically, PRRSv shows strong inhibitory effects on Type I interferons (IFN-β, IFN-α), affects the expression of several interleukins (i.e. IL-1, IL-6, IL-8, IL-10, TNF-α) and down-regulates swine leucocyte class I (SLA-I) antigens [10]. Also, several pattern recognition toll-like receptors (TLR3, TLR7, TLR8 and TLR9) are involved in the interaction between PRRSv and the host immune system [11]. It is increasingly evident that different strains may have a different potential for interfering with the pig immune system [10, 12]. Besides, host’s genetic traits may influence the immune response after PRRSv vaccination [13], or the course of PRRSv infection [14]. For instance, the risk of infection has been suggested to be related with several single nucleotide polymorphisms (SNPs) in CD163 and CD169 [15]. Also, other SNPs and microRNAs (miRs) especially in proteins involved in antiviral and inflammatory response have been associated to differential susceptibility to PRRSv infection [16–19].

In general, it is assumed that PRRSv only induces partial protection against heterologous strains [20] being impossible by now to forecast the degree of protection between two different isolates. In addition, large individual variation in the immune response is seen between individuals. For example, some individuals may show full or almost protection against a given isolate, while others are only partially protected or not protected at all [13].

The most widely admitted concept is that immunity against PRRSv is multigenic and may substantially vary between individuals. The aim of the present study was to compare several genes that encode for molecules involved in the immune response between two groups of vaccinated pigs that experienced short or long viremic periods after PRRSv challenge. These analyses include the sequencing of four SLA Class I, two Class II allele groups, and CD163 (Table 1), plus the analysis by quantitative realtime RT-PCR of the constitutive expression of TLR2, TLR3, TLR4, TLR7, TLR8 and TLR9 mRNA and other molecules in peripheral blood mononuclear cells (PBMC) (Table 2).

**Table 1:** **SLA Class I and Class II, allele groups and CD163 analysed by sanger sequencing**

Allele Group/gene	Amplicon size (in bp)	Contigs obtained	Mean coverage	PCR primers
SLA Class I				
SLA-1*07^a^	220	9	2	SLA-1*07XX F 5′-GCCGGGTCTCACACCATCCAGAT-3′
				SLA-1*07XX R 5′-GGCCCTGCAGGTAGCTCCTCAAT-3′
SLA-1*08^a^	577	18	2	SLA-1*08XX F 5′-CGCGTGGACTCCCGCTTCTTCATT-3′
				SLA-1*08XX R 5′-CCAGGAGCGCAGGTCCTCGTT-3′
SLA-2*05^a^	544	19	2	SLA-2*05XX F 5′-CGAGTGAACCTGCGCACAGCTCTT-3′
				SLA-2*05XX R 5′-CTGCAGCGTGTCCTTCCCCATCTC-3′
SLA-3*04^a^	192	20	2	SLA-3*04XX F 5′-GGAAGCCCCGTTTCATCGAA-3′
				SLA-3*04XX R 5′-GCAGGTTTTTCAGGTTCACTCGGA-3′
SLA Class II				
SLA-2*DQA^b^	898	20	3.38	DQAiF3 5′-CTAGAGACTGTGCCACAGATGAAG-3′
				DQAe3R1 5′-ACAGATGAGGGTGTTGGGCTGA-3′
SLA-2*DRB^c^	1308	12	2.51	DRBi1F9 5′-GCGGTGCCTTCAGCCTTTTCAGGAG-3′
				DRBi2R9 5′-AACAGTAGCAACTGTTTTGAGAGC-3′
CD163^d^	3813	11	3.55	CD163-Ex7Fw 5′-ATTCTGACTTCTCTCTGGAGGC-3′
				CD163-2700R 5′-GAGATGATGGGCACTGCCATAT-3′

Length of the PCR fragments amplified, number of contigs obtained (out of 20 analysed), mean coverage per position of the contigs obtained and primers used.

^a^[27], ^b^ [28], ^c^ [29], ^d^ [16].

**Table 2:** **Genes analysed by RT-qPCR. Length of the PCR fragments amplified, and primers used**

Gene	Ensemble gene	Amplicon size (bp)	PCR primers
TLRs			
TLR2^a^	ENSSSCG00000009002	162	TLR2-F 5′-TCACTTGTCTAACTTATCATCCTCTTG-3′
			TLR2-R 5′-TCAGCGAAGGTGTCATTATTGC-3′
TLR3^a^	ENSSSCG00000015801	112	TLR3-F 5′-AGTAAATGAATCACCCTGCCTAGCA-3′
			TLR3-R 5′-GCCGTTGACAAAACACATAAGGACT-3′
TLR4^a^	ENSSSCG00000005503	108	TLR4-F 5′-GCCATCGCTGCTAACATCATC-3′
			TLR4-R 5′-CTCATACTCAAAGATACACCATCGG-3′
TLR5^a^	Gene ID 100144476	156	TLR5-F 5′-CTCGCCCACCACATTA-3′
			TLR5-R 5′-TGAGGGTCCCAAAGAGT-3′
TLR7^a^	ENSSSCT00000035232.2		TLR7-F 5′-GGGAAAGCTCCAGTATCTGC-3′
			TLR7-R 5′-TGAGGCTTCTGGAACAGTTG-3′
TLR8^a^	ENSSSCG00000012118	105	TLR8-F 5′-AAGACCACCACCAACTTAGCC-3′
			TLR8-R 5′-GACCCTCAGATTCTCATCCATCC-3′
TLR9^a^	ENSSSCG00000011436	122	TLR9-F 5′-CACGACAGCCGAATAGCAC-3′
			TLR9-R 5′-GGGAACAGGGAGCAGAGC-3′
TNF-α^a^	ENSSSCG00000001404	102	TNF-α-F 5′-TGGTGGTGCCGACAGATGG-3′
IFN-γ^a^	ENSSSCT00000055560.1	132	TNFG-F-5′-CTGGGAAACTGAATGACTTCG-3′ TNFG-R-5′-TCTGACTTCTCTTCCGCTTTC-3′

^a^[30].

### Animal experiment

Samples selected come from animals studied in a transmission by contact experiment of PRRSv-1 [21]. Briefly, commercial cross-breed pigs were vaccinated with a live modified commercial vaccine and challenged with the PRRSv-1 strain CReSA3267 (Accession Number JF276435) 30 days later. Animals were bled at several time points before and after challenge. The viral load was quantified in sera samples by means of one step RT-PCR (qRT-PCR) targeting PRRSv ORF7. According to the duration of the viremia, 20 pigs were chosen and classified for the purposes of the present study as short-viremic (namely less than 5 days of viremia after challenge, SV, *n* = 8) or long-viremic (LV, ≥ 5 days of viremia, *n* = 12). The spleens and PBMCs of the selected animals collected at the end of the study (30 days post-challenge) were used for sequencing, TLR and cytokine expression analyses.

### Sanger sequencing

Table 1 depicts the host genes analysed and the primers used. Total DNA from spleen samples was extracted using the DNeasy Blood & Tissue Kit (QIAGEN, Hilden, Germany). PCR products were amplified using the AccuPrime™ Pfx DNA polymerase (Fisher Scientific, Hampton, USA) following the guidelines and the PCR conditions recommended by the supplier and the references provided. PCR products were sequenced using BigDye (Applied Biosystems, Foster City, USA) and chromatograms were aligned with SeqMan v7 (DNASTAR, Madison, USA). Mean coverages of the final contigs ranged between 2 and 3.55 lectures per position. Double peak ambiguities in heterozygote individuals were assigned to individual alleles by comparing them with the chromatograms of the homozygote ones. Thus, for every studied animal and gene analysed two alleles were built up. The evolutionary relationships among alleles were analysed by means of a Neighbor-Joining (NJ) tree based on the pairwise distance matrix, calculated with the Tamura-Nei model. The confidence of the tree internal branches were calculated with 1000 bootstrap pseudo-replicates.

**Figure 1:**
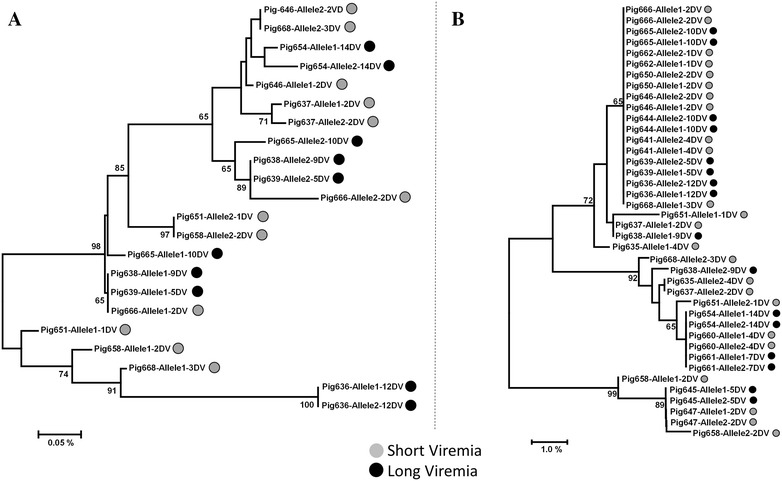
**Phylogenetic trees.** (**A**) NJ tree based on the 3813 bp of the CD163 analysed, corresponding to the exons 7–11 of the gene; (**B**) NJ tree based on the 641 bp of the SLA-1*04. Only bootstrap values higher than 65 were shown. Sequence labels include the pig identifier, the allele and the days of viremia.

All trees, irrespective of being calculated from the four SLA-Class I allele groups, the two SLA-Class II, or the CD163 datasets, showed a mixed clustering of SV and LV animals. Figure 1 shows as an example the phylogenetic trees obtained for the CD163 and the SLA-1*04 allele group. Moreover, in the present study, the three CD163 SNPs associated with PRRSv risk infection by [15] did not showed differential results between SV and LV groups: for the SNP A2552G the combination AA, associated with lower infections risk, was present in all but one pig; the higher risk genotype for the SNP G2277A was not present in any of the studied pigs; and in the SNP C2700A the higher risk genotype AA was present in a single individual, the same that not harboured the lower risk AA genotype for the A2552G SNP.

### qRT-PCR

**Figure 2:**
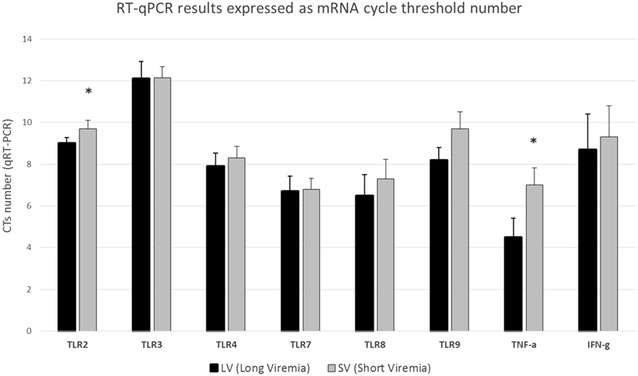
**qRT-PCR mRNA quantification.** Statistical differences in the cycle number of qRT-PCR amplification (CTs) of different Toll-like receptors (TLR) and other molecules involved in the immune response of the host between Long (LV) and Short (SV) viremic pigs. All Ct values were normalized with a housekeeping gene. *p < 0.05.

The constitutive levels of mRNA expression of different TLRs, TNF-α and IFN-γ were analysed in PBMCs (Table 2). Total RNA was extracted using Trizol (Invitrogen, Paisley, UK) according to the manufacturer’s instructions. To reduce DNA contamination, DNase digestion was conducted, followed with a further purification step using the RNeasy miniki (Qiagen, Crawley, UK). RNA samples were processed by a qRT-PCR one step (Brilliant III Ultra-Fast SYBR Green QRT-PCR Master Mix, Agilent technologies) with a Stratagene MX3000P (Stratagene, La Jolla, CA, USA). Samples were tested in triplicate, B-actin served as the housekeeping gene and results were calculated as described previously [22]. Before comparing groups all Ct values were normalized with the corresponding values of the housekeeping gene. Figure 2 shows the mean and SD cycle threshold number as a measure of the mRNA expression. Significant differences between groups were observed for TNF-α and TLR2 (*p* < 0.05); while a trend was observed for TLR8 and TLR9 (*p* = 0.06). In all these cases, the mean Ct values observed for the SV group were higher compared to the LV group, indicating a lower mRNA expression in pigs with shorter viremic periods.

## Discussion

The available literature about the genetics of host response to PRRSv show that several genes are involved in differential responses against the infection. Significant variation exists for a number of immune traits that at least include: antibody response, proliferative and cytokine responses of mononuclear cells, delayed-type hypersensitivity reactions, leukocyte number, and differential white blood cell counts (reviewed in [23]). Indeed, intrinsic immune differences have been reported among pig breeds [18, 22, 23], within herds [13] and even among tissues within an individual.

The results obtained in this study indicate a lack of correlation between the length and titre of the viremia, in vaccinated pigs and the clustering of the sequences of CD163, four SLA class I and two SLA class II allele groups (Figure 1). However, it should be noted that the low number of SLA allele groups analysed in the present study may certainly underrepresent the existing diversity. One of the most remarkable features of the SLA region is the extremely high degree of genetic polymorphism. More than a hundred allele groups have been defined for both SLA I (129) and SLA II (167); each of which present several alleles. For instance, 44 alleles have been described among the 12 SLA-1 allele groups; while, the 14 DRB1 allele groups present a total of 82 alleles [24]. Similarly, the amount of nucleotide diversity detected for the CD163 dataset (34 variable positions along the 3813 bp analysed) was not correlated with the length of the viremia (Figure 1). Also, none of the CD163 SNPs associated to PRRSv risk infection [15] were differentially expressed between SV and LV animals. Recent results from CD163 knockout pigs [25] indicate the complete absence of PRRSv-1 infection in PAMs and a substantial reduction in PRRSv-2, suggesting the pivotal role of CD163 in PRRSv-1 infection that may not be necessarily related with the length of the viremia. Finally, the constitutive mRNA expression levels of most analysed TLR genes in PBMC did not report significant differences with the exception of TLR2 and TNF-α expression (Figure 2). Interestingly, PBMCs of pigs with SV presented a lower constitutive mRNA levels of TNF-α and TLR2 than pigs with LV. This finding is noteworthy as it could indicate that a more limited inflammatory response may be operating in these pigs since exacerbated inflammatory responses in PRRSV-infected pigs have been correlated with more persistent infections and a worse clinical resolution [26]. Generally speaking, the results does not support, for the genes examined, the existence of a clear allele combination or host genetic profile that can be correlated with the length of the viremia in vaccinated animals.
